# Chemokine profile in women with moderate to severe anxiety and depression during pregnancy

**DOI:** 10.1186/s12884-021-04225-2

**Published:** 2021-12-04

**Authors:** Ignacio Camacho-Arroyo, Mónica Flores-Ramos, Ismael Mancilla-Herrera, Fausto Moisés Coronel Cruz, Joselin Hernández-Ruiz, Gabriela Pellón Diaz, Blanca Farfán Labonne, María del Pilar Meza-Rodríguez, Philippe Leff Gelman

**Affiliations:** 1 Unidad de Investigación en Reproducción Humana, Instituto Nacional de Perinatología-Facultad de Química, Universidad Nacional Autónoma de México, (CD MX) 04510 Mexico City, Mexico; 2 Instituto Nacional de Psiquiatría, CD MX 14370 Mexico City, Mexico; 3 Consejo Nacional de Ciencia y Tecnología/CONACyT, CD MX 03940 Mexico City, Mexico; 4 Departamento de Neurociencias, Instituto Nacional de Perinatología, Av. Montes Urales # 800. Col. Lomas de Virreyes, CD MX 11000 Mexico City, Mexico; 5 Clinical Pharmacology Unit, Hospital General de México Dr. Eduardo Liceaga, CD MX 06720 Mexico City, Mexico; 6 División of Nephology and Hypertension, University of Utah, Salt Lake City, UT 84112 USA

**Keywords:** Chemokines, Inflammation, Immune response, Pregnancy, Depression, Anxiety

## Abstract

**Background:**

Cytokine levels have been extensively described in pregnant subjects under normal and pathological conditions, including mood-related disorders. Concerning chemokines, very few studies have reported their association with psychiatric disorders during pregnancy. Therefore, we explored the chemokine profile in women exhibiting anxiety and depression during late pregnancy in the present study.

**Methods:**

One hundred twenty-six pregnant women in the 3rd trimester of pregnancy, displaying moderate to severe anxiety (ANX) alone and women exhibiting moderate to severe anxiety with comorbid depression (ANX + DEP), and 40 control pregnant women without affective disorders (CTRL) were evaluated through the Hamilton Anxiety Rating Scale (HARS) and the Hamilton Depression Rating Scale (HDRS). Serum chemokine levels of MCP-1 (CCL2), RANTES (CCL5), IP-10 (CXCL10), Eotaxin (CCL11), TARC (CCL17), MIP-1α (CCL3), MIP-1β (CCL4), MIG (CXCL9), MIP-3α (CCL20), ENA-78 (CXCL5), GROα (CXCL1), I-TAC (CXCL11) and IL-8 (CXCL8)] were measured by immunoassay. Clinical, biochemical, and sociodemographic parameters were correlated with HARS and HDRS score values.

**Results:**

Serum levels of most chemokines were significantly higher in the ANX and in the ANX + DEP groups, when compared to the CTRL group. Positive correlations were observed between MIP-1α/CCL3, MIP-1β/CCL4, MCP-1/CCL2, MIP-3α/CCL20, RANTES/CCL5, Eotaxin/CCL11, and I-TAC/CXCL11 with high scores for anxiety (HARS) (*p* < 0.05) and for depression (HDRS) (*p* < 0.004). After controlling clinical measures for age + gwk + BMI, chemokines such as IL-8/CXCL8, MCP-1/CCL2 and MIP-1β/CCL4 were found associated with high scores for anxiety (*p* < 0.05) in the ANX group. TARC/CCL17 and Eotaxin/CCL11 showed significant associations with high scores for depression (*p* < 0.04) whereas, MCP-1/CCL2 and MIP-1α/CCL3 were significantly associated with high scores for anxiety (*p* < 0.05) in the ANX + DEP group. Using a multivariate linear model, high serum levels of MIP-1β/CCL4 and Eotaxin/CCL11 remained associated with depression (*p* < 0.01), while, IL-8/CXCL8, MIP-1β/CCL4, MCP-1/CCL2, and MIP-1α/CCL3 were associated with anxiety (*p* < 0.05) in the symptomatic groups.

**Conclusions:**

Our data show that serum levels of distinct chemokines are increased in women exhibiting high levels of affective symptoms during late pregnancy. Our results suggest that increased levels of anxiety, depressive symptoms, and mood-related disorders may promote changes in specific functional chemokines associated with a chronic inflammatory process. If not controlled, it may lead to adverse obstetric and negative neonate outcomes, child development and neuropsychiatric alterations in the postnatal life.

**Highlights:**

Chemokine levels increase in affective disorders during pregnancy*.*

## Introduction

Major depressive disorder (MDD) is a debilitating condition with a high prevalence and multisymptomatic nature, representing the third cause of disability worldwide [1, 2]. MDD has a complex and multifactorial etiology that arises from complex interactions among genetic, developmental, and environmental factors, reflecting the heterogeneity of the disorder [3]. Such heterogeneity is reflected in the estimates of MDD individuals that receive antidepressant treatment, showing that only a third of patients receive adequate treatment, and up to half of them relapse despite the increasing number of antidepressant drugs currently available [2, 3]. Psychosocial stress and systemic disease can both affect the onset of depression, as shown for the comorbidity of depression in patients with diabetes, cancer, or cardiac disease, which is 17–29%, a percentage range much higher than MDD itself in the general population (10.3%) [3].

Current evidence suggests that the prevalence of depression in women during pregnancy is higher than the prevalence observed in similarly aged non-pregnant women [4]. Different studies reported that the prevalence of perinatal depression ranges between 2 and 21% [5, 6], increasing to 31% upon the self-report scales used to screen the healthy pregnant population [6]. A systematic review reported that prevalence rates for depression during pregnancy were 7.4, 12.8, and 12.0% for the first, second, and third trimesters, respectively [5, 6], whereas the prevalence of prenatal anxiety was reported between 15.8 and 25% [7, 8] and from 13 to 31.7% in the postpartum period [9]. Worth note is that the co-morbidity of both affective symptoms was 9.5% during pregnancy and 7.6% in the postpartum [10], and the impact of affective symptoms during the perinatal period included obstetric and neonatal adverse outcomes, family dynamic consequences, child morbidity, and mortality, medical complications among other issues in the postnatal life [11].

Immune and endocrine systems have been reported to be altered in pregnant women with affective disorders [12, 13]. Pregnant women with severe anxiety displayed higher cortisol levels than women without anxiety; conversely, dehydroepiandrosterone-sulphate (DHEA-S) levels were found significantly lower in women with high levels of anxiety when compared to healthy controls [14]. In a similar vein, previous studies showed that women exhibiting anxiety and comorbid depression in the third trimester of pregnancy exhibited significant increases of Th1 and Th17-related cytokines and higher ratios of Th1: Th2 immune balance, which correlated with high scores for anxiety and depressive symptoms [15]. These data suggest that variations in cytokine concentrations are likely influenced by the intensity of depressive and anxiety symptoms. Such inflammatory process could start escalating in vulnerable subjects with high anxiety levels, becoming conspicuous as severe depression emerges in pregnant women [16].

Other crucial immune mediators are the chemotactic cytokines or chemokines. These immune mediators participate in controlling cell migration and cell positioning during development, homeostasis, and inflammation. The common function of chemokines is characterized by the direct movement of leukocytes, the increased immune activity, and leukocyte recruitment [17].

The chemokine family consists of ≅ 50 endogenous ligands that bind to distinct rhodopsin-like seven-transmembrane-spanning receptors identified in humans and mice [18]. Chemokines are small, 8- to 12 kDa protein ligands that promote increased motility and directional migration after binding their cell-surface receptors. Chemokines and their gradients are detected upon signaling pertussis toxin-sensitive Gi-type G protein-receptors on target cells [18, 19].

Based on their function, chemokines are classified as follows: a) *Th1, CD8 and NK trafficking*-related chemokines: IP-10/CXCL10, I-TAC/CXCL11, and MIG/CXCL9; b) *Neutrophil trafficking-related chemokines:* IL-8/ CXCL8, ENA-78/CXCL5, and GROα/CXCL1; c) *Macrophage-NK migration/ lymphoid tissue T cell/DC interaction-related chemokines*: RANTES/CCL5, MIP-1α/CCL3, MIP-1β/CCL4; d) *Monocyte/macrophage trafficking-related chemokine:* MCP-1/CCL2*; e) Th17 response/B cell/DC homing to gut-associated lymphoid tissue:* MIP-3α/CCL20; f) *Th2 response/Th2 cell migration/Treg, lung and skin-homing:* TARC/CCL17; and g) *Eosinophil and basophil migration:* Eotaxin/CCL11 [18].

Chemokines have been implicated in neurobiological processes relevant to psychiatric disorders, such as synaptic transmission and plasticity, neurogenesis, and neuron-glia communication [20–22]. Disruption of these functions by activating the inflammatory response has been implicated in the pathogenesis and development of MDD [23, 24]. Chemokines promote innate and adaptive immune systems interactions, thus shaping and providing the necessary context for developing optimal adaptive immune responses [17].

Animal studies showed that an impaired chemokine/receptor -CXCL12/CXCR4- signaling leads to abnormal development, proliferation, and migration of neural progenitor cells [25, 26] and implicated in the reduced neurogenesis in MDD [20, 21]. In the same line, chemokine receptor knockout mice, CCR6, and CCR7 displayed behavioral phenotypes related to psychiatric disorders [27]. Interestingly, clinical studies showed an increase in several chemokines such as CCL2/MCP-1 and CXCL8/IL-8 serum concentrations in MDD subjects [21, 28].

Clinical studies have demonstrated an increase in CCL5 and CCL2 serum levels in subjects displaying generalized anxiety disorder [29], chronic stress [30], and post-traumatic stress disorder (PTSD) [31]. In healthy hospital workers, anxiety scores were found inversely associated with levels of CCL2, CCL5, CCL11, and IL-6 [32]. Recent clinical studies in the perinatal period showed that the expression of several chemokines and their receptors appear to be altered in women exhibiting postpartum depression [33], showing an innate immune activation of pro-inflammatory mediators (IL-6, IL-15, CCL3, GM-CSF) in the peripartum in pregnant women exhibiting affective symptoms [34].

Recent studies showed that plasma LPS level was significantly increased in depressed mothers during their 8–12 weeks gestation and which were associated with higher plasma levels of the pro-inflammatory cytokine (TNF-α) and the chemokine MCP/CCL2) compared to healthy controls [35]. Thus, albeit of the several reports describing the functional activity of chemokines in early pregnancy or pregnant women with depression during the first weeks of gestation, no studies have shown a close relationship between affective disorders and functional chemokines during late pregnancy. Thereby, we described the association found between serum levels of functional chemokines in women exhibiting high levels of depression and anxiety symptoms during late pregnancy.

## Methods

### Design of the Study

We performed a cross-sectional study from 2014 to 2016, similar to the those reported for cytokine and cortisol studies (Leff-Gelman et al., 2020; Leff Gelman et al., 2019) and performed at the General Hospital of Mexico /OB-Gyn Department (HGM, Hospital General de Mexico, Dr. Eduardo Liceaga of Mexico, Mexico City) and the National Institute of Perinatology/OB-Gyn-Outpatient Control Unit (INPer, Mexico City). Approval from the Institution Ethical Committee was obtained before the beginning of the study (HGM, D1/14/112/04/072, 2014–2016). During the third trimester of gestation pregnant women attended at the OB-Gyn Control Outpatient Units from both institutions were asked to participate in the study. Patients who willingly accepted to participate in it, required a written informed consent before the study. At entry, all participants required third trimester lab tests (blood count, biochemical testing, urinalysis, thyroid function), 2D fetal ultrasound, and Doppler Monitoring (data not shown). All patients were either inhabitants of Mexico City or neighboring states.

### Participants

Women between 18 and 30 years old, coursing a healthy pregnancy, during the third trimester (28–40 gwks) were invited to participate in the study. During the initial intervention at the OB-Gyn Control Outpatient Unit, a complete clinical and obstetric assessment was carried out, including sociodemographic parameters (marital status, education level, working status) and anthropometric measures (BMI, weight). Women were interviewed by the psychologist and asked to complete the self-reported questionnaire used to measure anxiety [Hamilton Anxiety Rating Scale (HARS)]. Patients exhibiting a score ≥ 25 in the HARS scale were considered with moderate to high intensity of anxiety symptoms, whereas patients with a cut-off score ≤ 5 were considered as healthy subjects and used as controls. In the same line, women were asked to complete the hetero-reported questionnaire used to measure depression [Hamilton Depression Rating Scale (HDRS)]. Patients exhibiting a score ≥ 24 in the HDRS scale, were considered with high levels of depressive symptoms, whereas patients with a cut-off score ≤ 7 were considered healthy controls. Women recruited into the study showed no smoking, alcohol consumption or drug abuse, including other associated mental (i.e., bipolar disorder, schizophrenia, psychosis, and neuropsychiatric pathologies (i.e., Alzheimer’s, seizures, attention deficit disorder, eating and uncontrolled compulsive disorders).

Exclusion criteria considered the following issues; patients receiving psychotropic medication, illicit substance use, patients having previous psychiatric diagnosis, obstetric pathologies (diabetes, hypertension, preeclampsia), infections, and medical illnesses (neurological, metabolic, cardiovascular, degenerative, endocrine, immune, and rheumatic disorders).

Moreover, participants were excluded from the study upon the demonstration of incomplete questionnaires, absence, abnormal findings in lab tests results and/or incomplete laboratory tests, and inconsistencies in the evaluation of the mood-related disorders.

Upon completing both anxiety and depression scale-related questionnaires, participants exhibiting moderate to high rating scores for anxiety with or without depression were referred to the psychiatric department for mood-disorder management and treatment. After the psychiatry interview and completion of the clinician-rated questionaries, patients were remitted back to the Ob-Gyn outpatient unit to proceed for blood sampling and quantification of serum chemokines.

### Criteria and clinician-rated instruments

Criteria considered for recruitment of patients was the presence of high levels of anxiety following the 14 item-Hamilton Anxiety Rating Scale (HARS), which assesses the intensity of anxiety symptoms by a 5-point Likert scale ranging from 0 (no symptom) to 4 (severe anxiety), and higher total scores indicate severe anxiety [36]. Thus, HARS scores are categorized as follows: mild anxiety (score 7–17), moderate anxiety (score 18–24), severe anxiety (score > 25). Patients with a total score > 25 were considered as subjects with moderate to high levels of anxiety [36].

Depressive symptoms were evaluated using the 17-Item Hamilton Depression Rating Scale (HDRS) [37]. The Hamilton Rating Scale for Depression is a multidimensional and clinician-rated scale that has become the standard for clinical trials of depression. For the 17-item version of the Hamilton Rating Scale for Depression, scores can range from 0 to 54. It is generally accepted that scores between 0 and 6 indicating the absence of depression, scores between 7 and 17 indicate mild depression, scores between 18 and 24 indicate moderate depression and scores over 24 indicate severe depression [38, 39]. Therefore, patients with a cutoff > 24 were considered as subjects with moderate to high levels of depression.

Both HDRS and HARS instruments were applied to healthy women to confirm the absence of depressive and anxiety symptoms. Thus, patients showing a cut-off score < 5 in the anxiety-rating scale and a cut-off score < 7 in the depression-rating scale were considered the control group. All patients were evaluated by psychiatric interview following the Diagnostic and Statistical Manual of Mental Disorders (DSM-5; American Psychiatric Association, 2015). Both instruments were validated in the local language [37, 40].

The anxiety scale used in the study has been extensively used to assess the intensity of mood- related items such as, subjective feelings, autonomic and somatic symptoms, cognitive functions, and behavioral responses. In the same line, the depression scale used herein has been widely used to assess the intensity of depressive mood, suicide thought or action, insomnia, irritability, fears, somatic symptoms, libido dysfunction, cognitive function, and insight [41, 42]. Both scales were shown to be reliable, specific, and sensitive clinician-rated instruments [43]. Rating scores obtained from each instrument were checked by the Psychiatric Department, and final evaluations were recorded in a clinical database. Patients exhibiting moderate to higher levels of anxiety and depression symptoms were referred to the psychiatry department for mood-disorder evaluation and treatment.

All participants were screened for depressive and anxiety symptoms, clinical variables, sociodemographic parameters, anthropometric measures.

### Screening, recruitment and study groups

**Fig. 1:**
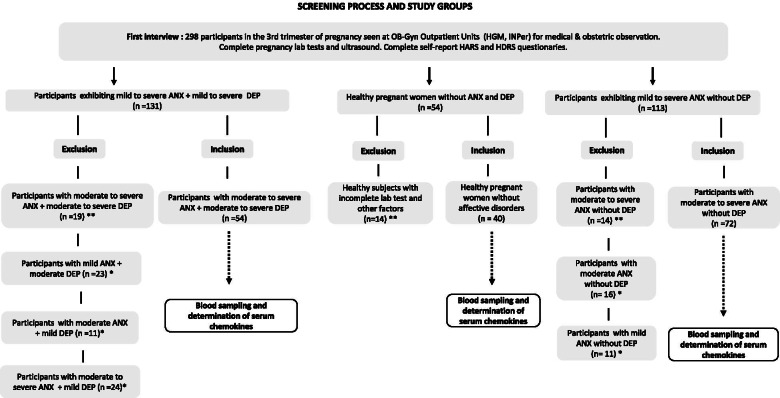
Screening and recruitment process of participants into the study. The figure depicts the screening and recruitment process of participants with affective symptoms and healthy pregnant women during the third trimester of pregnancy at the beginning of the study. Both inclusion and exclusion criteria were used to define the study groups formed at the end of the study. (* **) Some participants were excluded due to incomplete or abnormal lab tests, inconsistencies in questionaries, absence to medical/lab appointments, resign or quit from the study (see methods)

We screened a total of 298 participants at the Ob-Gyn outpatient units at the beginning of the study, and who showed mild to severe anxiety with or without mild to severe depression (*n* = 131), women with mild to intense levels of anxiety symptoms without depression (*n* = 113), and healthy pregnant women displaying no affective disorders (*n* = 54) in the 3rd trimester of pregnancy (see flow diagram, Fig. 1).

Clinical measures, sociodemographic parameters, depressive and anxiety symptoms and other comorbid psychiatry disorders were assessed during patients’ interview. Participants not fulfilling with the items depicted in the inclusion criteria were formaly excuded fom the study (see inclusion and exclusion criteria above). Thus, after the initial screening and elimination process of participants, a total of 166 patients were finally recruited into the study and clustered into three different groups; a) pregnant women exhibiting moderate to severe anxiety without depression (ANX, *n* = 72); b) pregnant women displaying moderate to severe anxiety and comorbid (moderate to severe) depression (ANX+ DEP, *n* = 54); and c) healthy pregnant women without affective disorders, used as controls (CTRL, *n* = 40).

### Blood sampling and quantification of serum chemokines

Blood sampling was performed at the main clinical lab facility under aseptic conditions from 7:00–9:00 am, following standardized laboratory procedures for blood extraction and collection. Samples from 176 pregnant women with 8–12 h fasting conditions were collected and processed as described below.

Heparinized tubes (BD, USA) containing blood samples were handled on ice for 30 min and then centrifuged at 1000–2000 x g for 10 min in a Refrigerated Eppendorf Centrifuge 5702R (Eppendorf, USA). Serum fractions were separated and aliquoted into 1.5 mL cryogenic vials and stored at − 70 °C until further use, as previously described [15]. Cryogenic vials were numbered in sequence following the patient’s clinical file and date of sample processing.

Serum chemokines [MCP-1 (CCL2), RANTES (CCL5), IP-10 (CXCL10), Eotaxin (CCL11), TARC (CCL17), MIP-1α (CCL3), MIP-1β (CCL4), MIG (CXCL9), MIP-3α (CCL20), ENA-78 (CXCL5), GROα (CXCL1), I-TAC (CXCL11) and IL-8 (CXCL8)] were quantified by an immunoassay based on multiplex bead array using the LEGENDplex™ Human Proinflammatory Chemokine Panel (Cat. 740,003, BioLegend, USA) following manufacturer’s instructions. Briefly, serum samples (50 μL) were thawed and incubated with specific chemokine-antibody coated beads in a mix buffer at room temperature for 1.5 h in the presence of specific fluorochrome per chemokine. After several washes, samples were analyzed in a FACS Aria III flow cytometer (BD, USA). The chemokine levels were calculated using the LEGENDplexTM Data Analysis Software v 7.0 (Biolegend, CA, USA). The lower limits of detection of each chemokine assayed (manufacturer ‘s data) were: IP-10/CXCL10 (1.4 pg/mL), I-TAC/CXCL11 (7.8 pg/mL), MIG/CXCL9 (9.4 pg/mL), IL-8/CXCL8 (1.4 pg/mL), ENA-78/CXCL5 (1.1 pg/mL), GROα/CXCL1 (3.2 pg/mL), RANTES/CCL5 (4.3 pg/mL), MIP-1α/CCL3 (2.1 pg/mL), MIP-1β/CCL4 (2.3 pg/mL)*,* MCP-1/CCL2 (0.9 pg/mL), MIP-3α/CCL20 (2.5 pg/mL), TARC/CCL17 (0.8 pg/mL) and Eotaxin/CCL11 (0.8 pg/mL). Intra-assay coefficient was < 3.0% and inter-assay covariance was < 5.0%.

Data obtained from flow cytometer analyses by lab observers were uploaded into a lab database. All data collected from patients were handled by personal involved in the protocol following a double-blind study. The number of cryogenic vials, serum chemokine concentration/patient, vacutainer-test tube and patient’s file number, were only known by the present study’s leading researchers.

### Statistical analysis

Serum levels of chemokines are shown as mean ± SEM. Clinical variables are depicted in percentage values (%). The parametric t-test with Welch’s correction (two-tailed *p*-value) was used to compare the means of chemokine serum levels among the study groups. Similarly, Pearson bivariate correlations were performed to assess the associations between biological variables, sociodemographic measures, and questionnaires-related scores among the study groups. Moreover, Pearson correlations were used to detect the associations between serum chemokines and clinical measures after controlling all variables for age, gwk and BMI in the study groups. In addition, a general linear model was used to detect the remaining associations between functional chemokines and affective symptoms in the tested groups. Two-way ANOVA was used to determine significant differences in clinical and biological measures among groups. In detecting significant differences between the parameters assayed in the study, a post hoc Tukey test was performed to establish the differences observed between serum chemokines and clinical variables among the tested groups. Statistical analyses were performed using GraphPad Prism 7 (GraphPad Softwares Inc. USA) and SPSS software v.27.0 (Armonk, NY: IBM Corp). For all the statistical analyses, the *p*-value ≤ 0.05 was considered significant.

## Results

### Demographic characteristics

Table 1 shows the sociodemographic characteristics of non-white Latin pregnant women (*n* = 166) recruited in the study in the 3rd trimester of pregnancy, presenting a mean gwk of 35.1 ± 1.3, and a mean age of 25.7 ± 2.1 years-old participating in the study. Control subjects were older than those corresponding to the anxiety (ANX) and the depressive (ANX+ DEP) groups, respectively. As shown, significant differences were found in the intensity of anxiety symptoms (HARS scores) among the studied groups (t-test, *p* = 0.000), and between age and gwk (t-test, *p* < 0.005) among the depressive and the control groups, respectively.

**Table 1:**
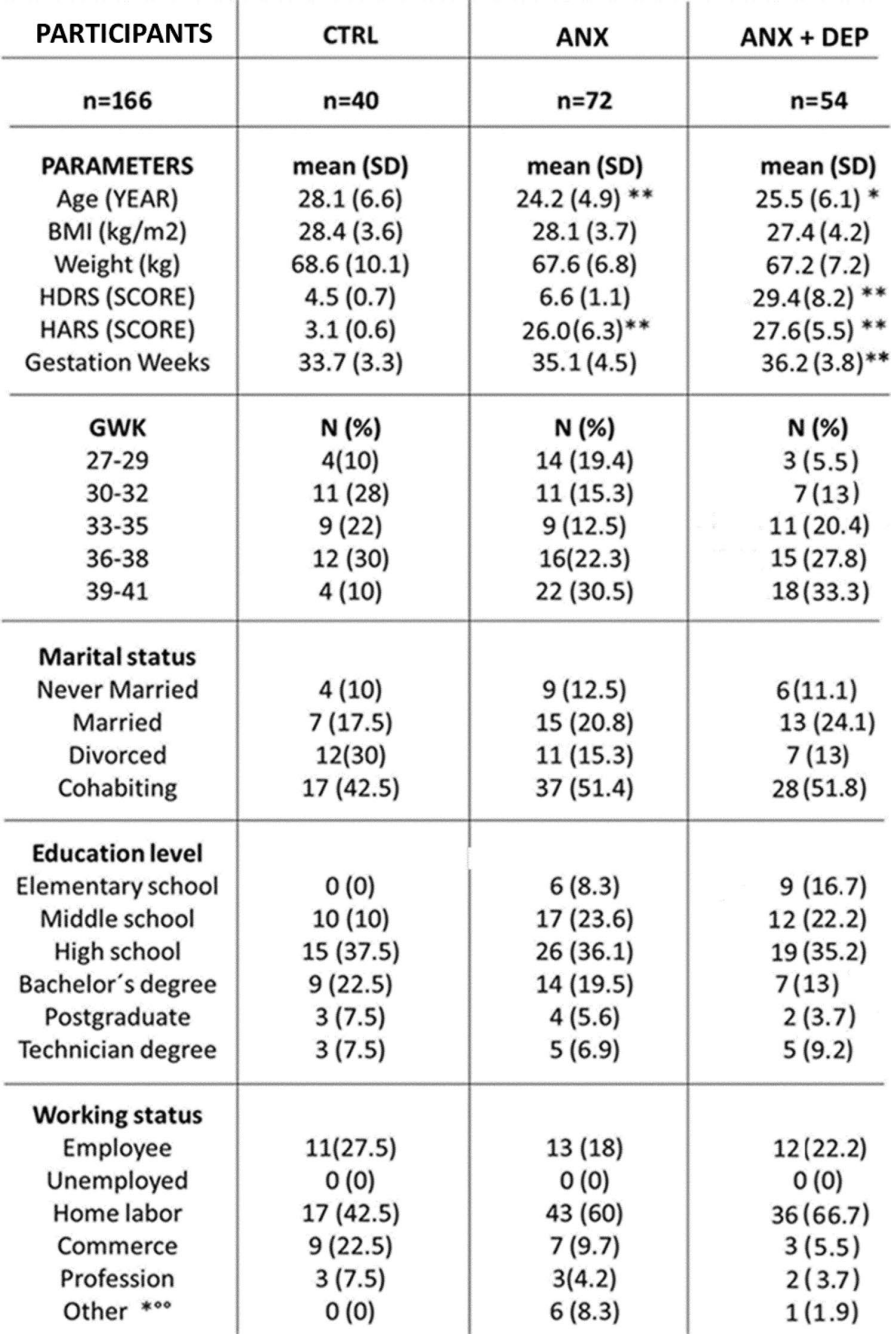
The parametric t-test with Welch’s correction was used to detect statistical differences between demographic measures among the studied groups. (*) *p* < 0.005 indicates the age differences found among tested groups. (**) *p* < 0.001, indicates the differences found between the final HDRS score values estimated for either the SA + SD and the CTRL groups, respectively; or between the final HARS score values estimated for each of the studied groups. Data are expressed as the mean ± SD. Data were calculated using GraphPad-v.7. Abbreviations: ANX + DEP, high anxiety plus comorbid depression; ANX, high anxiety; CTRL; control; HDRS, Hamilton Depression Rating Scale; HARS, Hamilton Anxiety Rating Scale; BMI, Body Mass Index; GWK, gestational weeks

### Serum chemokine levels

**Fig. 2:**
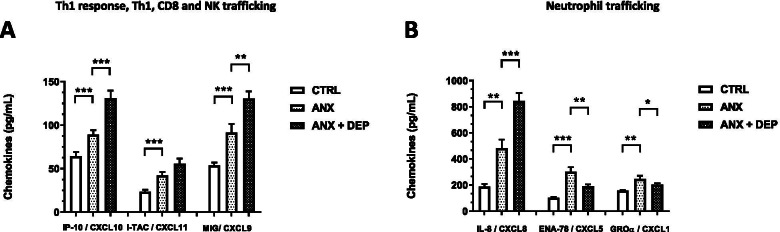
Concentrations of Th1, CD8, NK trafficking, and Neutrophil trafficking-related chemokines in the ANX, ANX + DEP and CTRL groups. The figures depict the values of the estimated serum concentrations of Th1, CD8, and NK trafficking-related chemokines, *IP-10/CXCL10, I-TAC/CXCL11, and MIG/CXCL9* (**A**), and the Neutrophil trafficking-related chemokines, IL*-8/CXCL8, ENA-78/CXCL5, and GROα/CXCL1* (**B**) in the CTRL, ANX, and ANX + DEP groups. These immune mediators (Th1, CD8, and NK trafficking- and Neutrophil trafficking-associated chemokines) were quantified by flow cytometry (see methods). Values are expressed as the mean ± SEM. The parametric, t-test analysis with Welch’s correction was used to estimate the *p* values for each functional chemokine assayed in the study among the tested groups. (*) *p* < 0.05; (**); *p* < 0.001; (***) *p* < 0.0001. ANX + DEP, high anxiety plus comorbid depression; ANX, high anxiety; CTRL, control. Data were calculated using GraphPad v.7

Regarding the *Th1/CD8/NK trafficking*-related chemokines, the serum levels of IP-10/CXCL10, I-TAC/CXCL11, and MIG/CXCL9 in the ANX and the ANX + DEP groups were significantly higher than those in the CTRL group. Besides, the serum levels of IP-10/CXCL10 and MIG/CXCL9 in the ANX + DEP groups were significantly higher than those in the ANX group (Fig. 2A). IP-10/CXCL10 and MIG levels were higher compared with those of I-TAC/CXCL11. The serum levels of the *Neutrophil trafficking*-related chemokine, IL-8/CXCL8, were higher in the ANX and the ANX + DEP groups than those in the CTRL group. Interestingly, ENA-78/CXCL5 and GRO-α/CXCL1 levels were higher in the ANX group compared with the other groups (Fig. 2B).

**Fig. 3:**
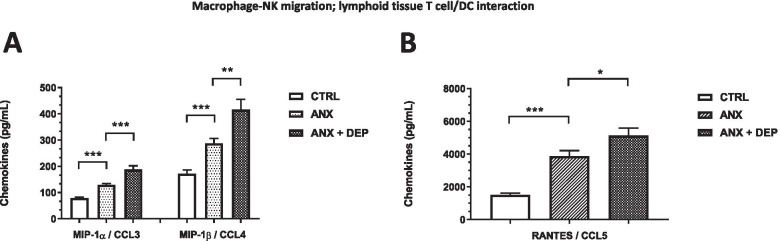
Concentrations of the Macrophage-NK migration, lymphoid tissue T cell/DC interaction-related chemokines in the ANX, ANX + DEP, and CTRL groups. The figures depict the values of the serum estimated concentrations of Macrophage-NK migration/ lymphoid tissue T cell/DC interaction-related chemokines, *MIP-1α/CCL3, MIP-1β/CCL4* (**A**) and *RANTES/CCL5,* (**B**) in the CTRL, ANX, and ANX + DEP groups. These immune mediators (Macrophage-NK migration, lymphoid tissue T cell, DC interaction-associated chemokines) were quantified by flow cytometry (see methods). The concentration values are expressed as the mean ± SEM. The parametric, t-test analysis with Welch’s correction was used to estimate the *p* values for each functional chemokine assayed in the study among the tested groups. (*) *p* < 0.05; (**); *p* < 0.001; (***) *p* < 0.0001. ANX + DEP, high anxiety plus comorbid depression; ANX, high anxiety; CTRL, control. Data were calculated using GraphPad v.7

The serum levels of the *Macrophage-NK migration/lymphoid tissue T cell/DC interaction*-related chemokines, MIP-1α/CCL3, MIP-1β/CCL4, and RANTES/CCL5 were significantly higher in the ANX and in the ANX + DEP groups when compared to their levels in the CTRL group. The highest levels of these chemokines were observed in the ANX + DEP group (Fig. 3A-B).

**Fig. 4:**
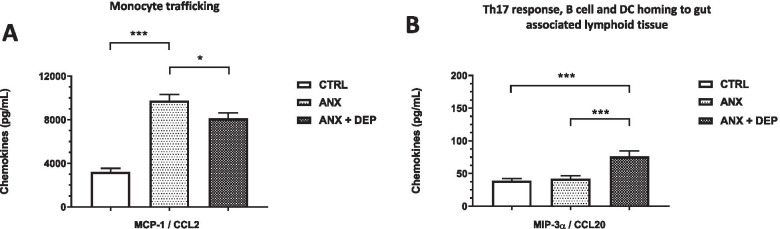
Concentrations of Monocyte trafficking- and Th17 response, B cell, DC homing to gut-associated lymphoid tissue-related chemokines in the ANX, ANX + DEP and CTRL groups. The figures depict the values of the serum estimated concentrations of Monocyte trafficking-related chemokine, *MCP-1/CCL2 (***A**) and the Th17 response, B cell, DC homing to gut-associated lymphoid tissue-related chemokine, *MIP-3α/CCL20* (**B**) in the CTRL, ANX, and ANX + DEP groups. These immune mediators (Monocyte trafficking- and Th17 response, B cell, DC homing to gut associated lymphoid tissue- chemokines) were quantified by flow cytometry. The concentration values are expressed as the mean ± SEM. The parametric, t-test analysis with Welch’s correction was used to estimate the *p* values for each functional chemokine assayed in the study among the tested groups. (*) *p* < 0.05; (**); *p* < 0.001; (***) *p* < 0.0001. ANX + DEP, high anxiety plus comorbid depression; ANX, high anxiety; CTRL, control. Data were calculated using GraphPad v.7

Concerning the monocyte trafficking chemokine, *MCP-1/CCL2,* its levels were higher in the ANX and the ANX + DEP groups than in the CTRL group. The highest levels were found in the ANX group. Concerning the *Th17 response, B cell, and DC homing to gut-associated lymphoid tissue*-*related chemokine*, the serum levels of MIP-3α/CCL20 were higher in the ANX + DEP group with regard to the other groups (Fig. 4A-B).

**Fig. 5:**
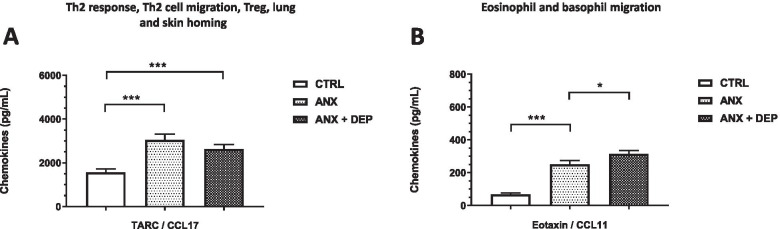
Concentrations of Th2 response, Th2 cell migration, Treg, lung and skin homing- and the Eosinophil and basophil migration-related chemokines in the ANX, ANX + DEP, and CTRL groups. The figures depict the values of the serum estimated concentrations of the Th2 response, Th2 cell migration and Treg, lung and skin homing-related chemokine, *TARC/CCL17* (**A**) and the Eosinophil and basophil migration-related chemokine, *Eotaxin/CCL11* (**B**) in the CTRL, ANX, and ANX + DEP groups. These immune mediators (Th2 response, Th2 cell migration and Treg, lung and skin homing- and Eosinophil and basophil migration-related chemokines) were quantified by flow cytometry. The concentration values are expressed as the mean ± SEM. The parametric, t-test analysis with Welch’s correction was used to estimate the *p* values for each functional chemokine assayed in the study among the tested groups. (*) *p* < 0.05; (**); *p* < 0.001; (***) *p* < 0.0001. ANX + DEP, high anxiety plus comorbid depression; ANX, high anxiety; CTRL, control. Data were calculated using GraphPad v.7

Serum levels of the *Th2 response, Th2 cell migration, Treg, lung, and skin homing*-*related chemokine*, TARC/CCL17, and the eosinophil *and basophil migration-related chemokine*, Eotaxin/CCL11, were higher in the ANX and the ANX + DEP groups than in the CTRL group, and the highest levels of TARC/CCL17 and Eotaxin/CCL11 were observed in the ANX and the ANX + DEP groups, respectively (Fig. 5A-B).

### Bivariate correlations between serum chemokines and clinical variables

Table 2 depicts the correlations between serum chemokine levels and clinical variables among the studied groups. As shown, all groups of patients recruited into the study displayed positive correlations with several chemokines, HARS and HDRS scores, and clinical variables.

**Table 2:**
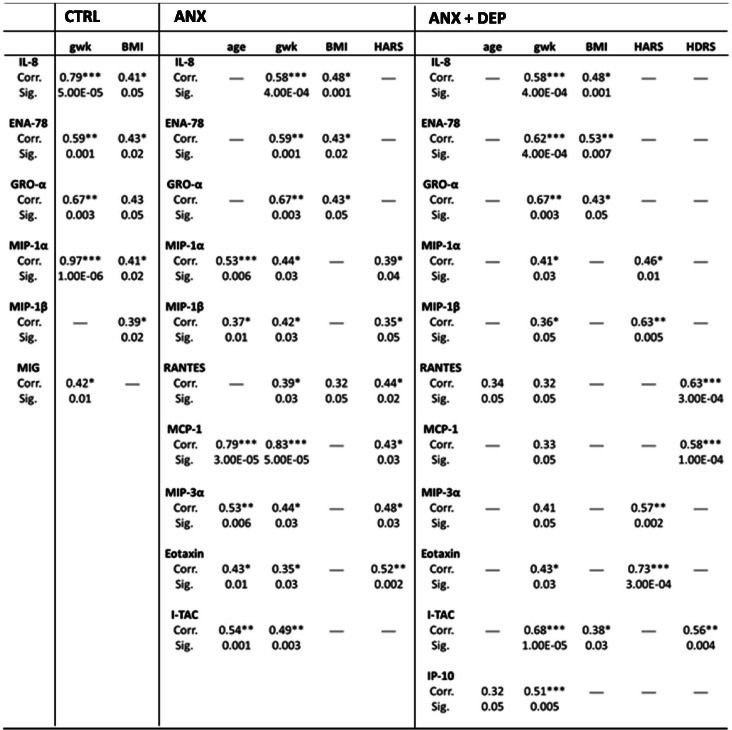
Bilateral correlations between chemokines, psychometric and demographic measures. SSPS software v.24.0 was used to determine the Pearson correlations among the ANX, ANX + DEP and the CTRL groups. Abbreviations: ANX + DEP, high anxiety plus comorbid depression; ANX, high anxiety; CTRL; control; HDRS, Hamilton Depression Rating Scale; HARS, Hamilton Anxiety Rating Scale; BMI, Body Mass Index; gwk, gestational weeks; Corr., correlation; Sig., significance. (*) Significant correlation at a *p* value < 0.05; (**) Significant correlation at a *p* value < 0.01; (***) Significant correlation at a *p* value < 0.001

As shown, only MIP-1α/CCL3, and MIP-1β/CCL4, MIP-3α/CCL20, MCP-1/CCL2, RANTES/CCL5, Eotaxin/CCL11 displayed positive correlations with HARS scores (Pearson, *p* < 0.04) and with clinical variables used in our study (Pearson; age, *p* < 0.01; gwk, *p* < 0.03; BMI, *p* < 0.05) in the ANX group. Moreover, other functional chemokines (I-TAC/CXCL11, IL-8/CXCL8, ENA-78/CXCL5, GRO-α/CXCL1) also showed related correlations with clinical measures (Pearson, *p* < 0.05).

In the same vein, MIP-1α/CCL3, MIP-1β/CCL4, MIP-3α/CCL20, and Eotaxin/CCL11 showed positive correlations with high levels of anxiety symptoms (HARS scores) (Pearson, *p* < 0.01) in the ANX + DEP group; whereas I-TAC/CXCL11, RANTES/CCL5, and MCP-1/CCL2 CCL11 displayed significant correlations with high HDRS scores (Pearson, *p* < 0.004), in addition of correlating with different clinimetric variables.

Other functional chemokines (IL-8/CXCL8, ENA-78/CXCL5, and Gro-α/CXCL1) (Pearson, *p* < 0.05) showed similar related associations with clinical variables in the ANX + DEP and CTRL groups (Pearson, *p* < 0.01), respectively.

Significant differences were found between MIP-1α/CCL3, MIP-1β/CCL4, and MIP-3α/CCL20 and HARS scores in both of the symptomatic groups (Tukey test, *p* < 0.05); whereas MIP-3α/CCL20 showed significant differences with HARS scores (Tukey test, *p* < 0.03) in the depressive group (data not shown).

### Partial correlations between serum chemokines and clinical variables

As depicted in Table 3, after adjusting clinical data for age, BMI and gwk, altogether in the ANX group, functional chemokines such asIL-8/CXCL8 (Pearson, *p* = 0.01), MCP-1/CCL2 (Pearson, *p* = 0.03), and MIP-1β/CCL4 (Pearson, *p* = 0.05) showed positive correlations with high levels for anxiety symptoms (HARS scores), whereas in the ANX + DEP group, MCP-1/CCL2 (Pearson, *p* = 0.01), TARC/CCL17 (Pearson, *p* = 0.02), Eotaxin/CCL11 (Pearson, *p* = 0.01), and MIP-1α/CCL3 (Pearson, *p* = 0.003) were found displaying positive correlations with high scores for depression symptoms (HDRS scores) (Pearson, *p* < 0.04) .

**Table 3:**
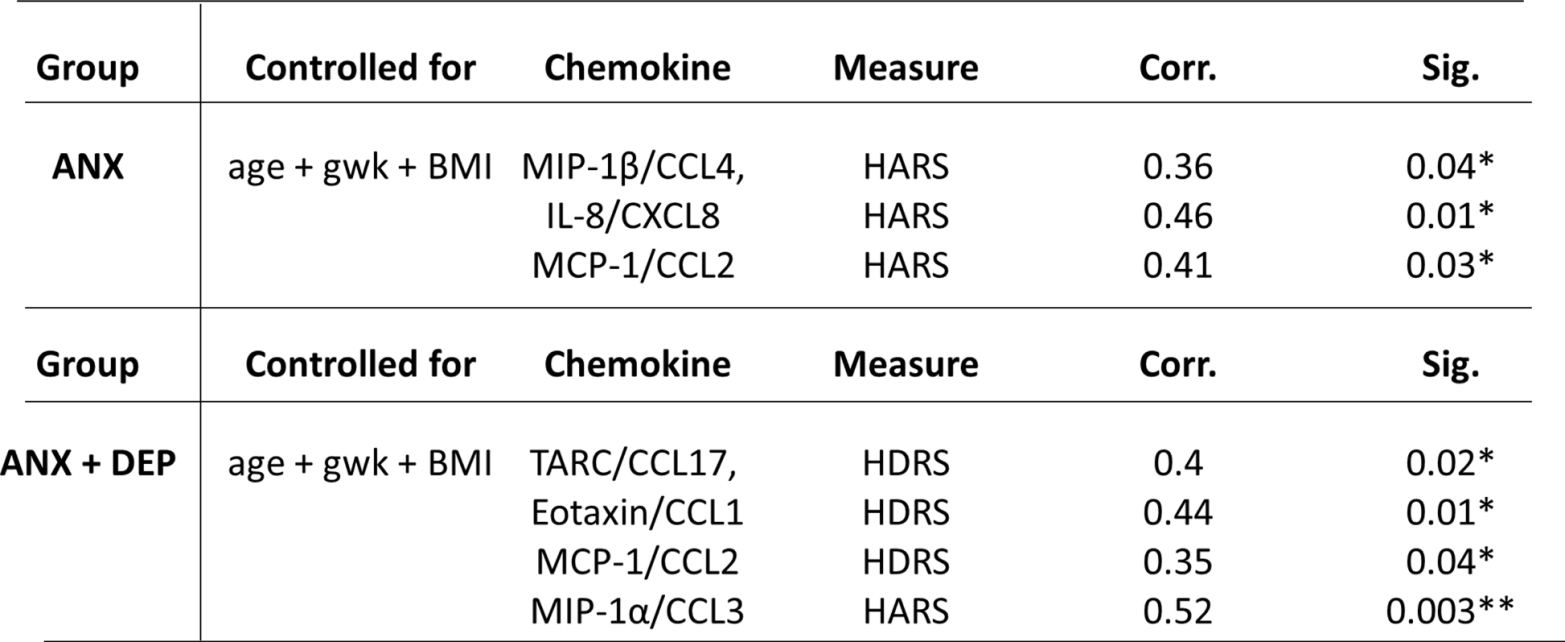
SSPS software v.24.0 was used to determine the partial correlations between clinical parameters and serum chemokines in the symptomatic groups. Correlations and *p* values were obtained after controlling clinical measures for *age + gwk + BMI* and, used as the dependent variables for the analysis (see text for details). Abbreviations: ANX + DEP, high anxiety plus comorbid depression; ANX, high anxiety; BMI, Body Mass Index; gwk, gestational weeks; HDRS, Hamilton Depression Rating Scale; HARS, Hamilton Anxiety Rating Scale; Corr., correlation; Sig., significance. (*) Significant correlation at a *p* value < 0.05; (**) Significant correlation at a *p* value < 0.01

### General lineal model

As shown in Table 4, using a multivariate linear model, serum levels of chemokines, such as IL-8/CXCL8 (F = 12.1, *p* = 0.003), MCP-1/CCL2 (F = 4.7, *p* = 0.04), MIP-1β/CCL4 (F = 5.7, *p* = 0.03) and MIP-1α/CCL3 (F = 4.4 *p* = 0.05) were found associated with high scores for anxiety symptoms (HARS) in the ANX group; whereas high serum levels of IL-8/CXCL8 (F = 4.5, *p* = 0.05) and MIP-1α/CCL3 (F = 4.7, *p* = 0.05) remained associated with high scores for anxiety symptoms in the ANX + DEP group, in addition of MIP-1β/CCL4 (F = 17.6, *p* = 0.004) and Eotaxin/CCL11 (F = 7.9, *p* = 0.01), which were associated with high scores for depressive symptoms (HDRS).

**Table 4:**
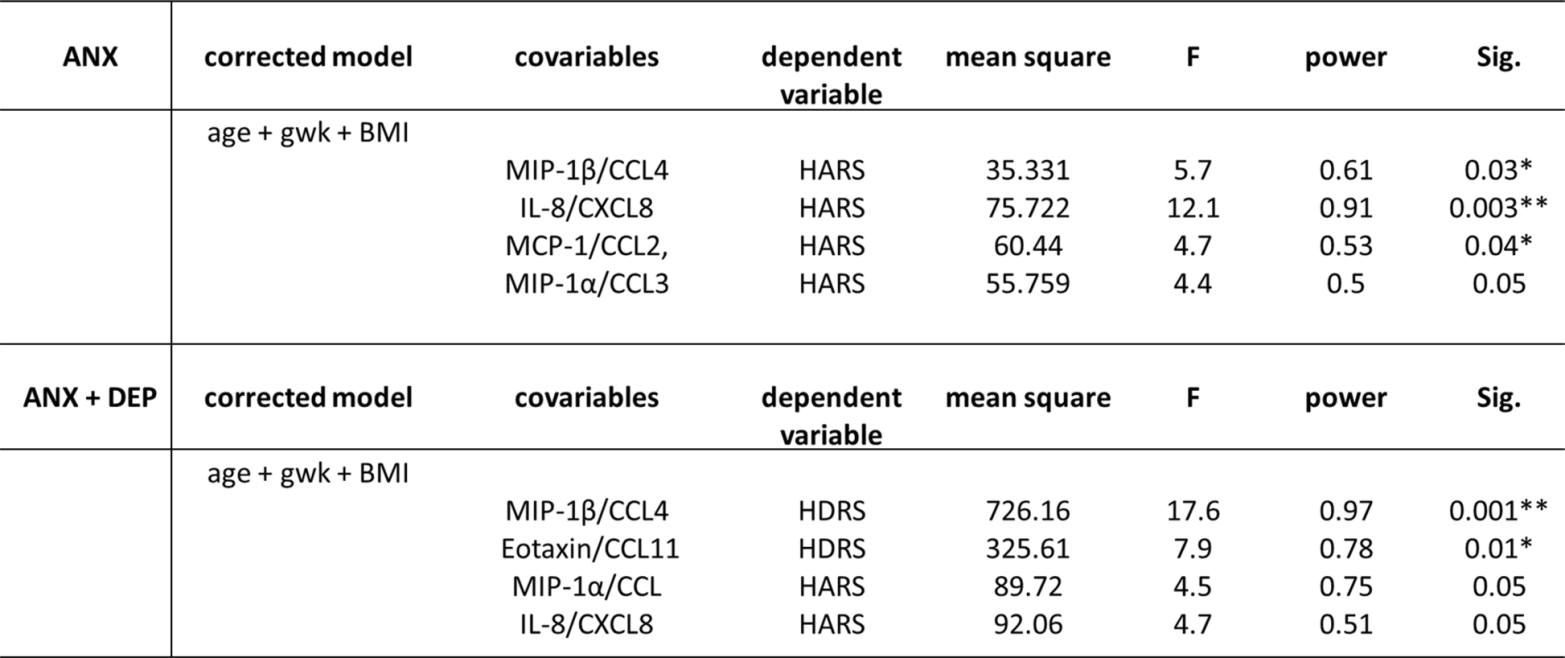
SSPS software v.24.0 was used to determine the remaining associations between clinical parameters and serum chemokines in the symptomatic groups. The statistical results described (mean square, F, power observed and *p* values) were obtained using the *multivariate general lineal model*, after controlling clinical data for *age + gwk + BMI* (see text for details). Abbreviations: ANX + DEP, high anxiety plus comorbid depression; ANX, high anxiety; BMI, Body Mass Index; gwk, gestational weeks; HDRS, Hamilton Depression Rating Scale; HARS, Hamilton Anxiety Rating Scale; Corr., correlation; Sig., significance. (*) Significant correlation at a *p* value < 0.05; (**) Significant correlation at a *p* value < 0.01

## Discussion

The present study shows that pregnant subjects exhibiting either higher levels of anxiety symptoms and/or comorbid anxiety and depression symptoms, showed higher concentrations of distinct serum chemokines than healthy pregnant women.

Recent clinical studies have focused on the role of functional chemokines in neuropsychiatric diseases [44], including their effects after antidepressant treatment [45]. Chemokines studied in affective disorders [21, 46, 47]; include the monocyte chemoattractant protein (MCP)-1/CCL2 [48–50]; the macrophage inflammatory protein (MIP)-1α/CCL3 [51–53]; Gro-α/CXCL1 [22, 54, 55]; The IL-8/CXCL8 [48, 56, 57]; TNF-β [58]; IL-16 [58]; CTACK [56]; macrophages migration inhibitory factor (MIF) [56, 59]; Eotaxin/CCL11 [60, 61]; CXCL11/ITAC [62]; MEC/CCL28 [63];TECK/CCL25 [63]; interferon gamma-induced protein (IP)-10/CXCL10 [64, 65]; RANTES/CCL5 [21, 66, 67].

Chemokines play crucial roles during the early stages of pregnancy, not only in the recruitment and functional regulation of decidual immune cells but also in the blastocyst/embryo implantation into the uterus and trophoblast invasion [68]. Several studies showed that chemokine/chemokine receptor interactions control leukocytes and decidual immune cells’ trafficking, recruitment, and maintenance [69]. Our results showed that a large number of chemokines (i.e., IP-10/CXCL10, I-TAC/CXCL11, MIG/CXCL9, RANTES/CCL5, MIP-1α/CCL3, MIP-1β/CCL4, MIP-3α/CCL20, including the IL-8/CXCL8 and Eotaxin/CCL11-related chemokines) were found significantly increased in patients exhibiting high levels of anxiety and depressive symptoms.

Similarly, chemokines such as ENA-78/CCL5, GROα/CXCL1, MCP-1/CCL2, and TARC/CCL17 were increased in women displaying high anxiety levels. Although, at present, it is uncertain how these chemokines are enrolled in affective disorders during pregnancy, recent reports highlight some of the negative effects produced by inflammatory chemokines during pregnancy, as shown for the high levels of inflammatory chemokines (i.e., IL-8, MCP-1, and MIP-1α) measured in the cord blood, and reported to enhance intrauterine inflammation, premature birth, and neonatal complications in perinatal women [70]. Such findings were also associated with other neonatal complications, such as patent ductus arteriosus, respiratory distress syndrome, and chronic lung disease [70].

Interestingly, recent studies showed that mothers of children with autism spectrum disorders (ASD) with intellectual disabilities (DQ < 70) (ASD + ID) had significantly elevated mid-gestational levels of cytokines (GM-CSF, IFN-γ, IL-1α, IL-6) and chemokines (IL-8, MCP-1) when compared to either mother of children with ASD without intellectual disabilities (ASD-noID, DQ ≥70) and mothers of the general population (GP) controls. Conversely, mothers of children with either ASD-noID or developmental delay (DD) had significantly lower levels of the chemokines IL-8 and MCP-1 than mothers of GP controls. Findings that suggested differences in the psychiatric disorders might be related to the plausible expression of early immune biomarkers specific to sub-phenotypes of ASD [71].

Different studies showed that the increased serum concentrations of MCP-1/CCL2, IL-8/CXCL8 in MDD subjects [21] correlated with the onset and progression of MDD (21, 24, 29). In the same line, related studies showed an increase in ENA-78/CCL5 and MCP-1/CCL2 serum levels in subjects displaying generalized anxiety disorder [29], chronic stress [30], and post-traumatic stress disorder [31]. Interestingly, our results are in line with the aforementioned data, showing that chemokines such as MCP-1/CCL2, TARC/CCL17, ENA-78/CXCL5, and GRO-α/CXCL1 were significantly increased in pregnant women exhibiting ANX.

MCP-1/CCL2 appears to be dysregulated in stress-inducing anxiety-like behaviors in humans and rodents [71–73]. Animal studies showed that Balb/C mice exposed to chronic ethanol consumption leads to an increase in chemokine levels (MCP-1, MIP-1α, CX3CL1) in the striatum and serum (MCP-1, MIP-1α, CX3CL1) [72]. Alcohol deprivation for 24 h induced IFN-γ levels in the striatum and maintained high levels of some cytokines (IL-1β, IL-17) and chemokines (MIP-1α, CX3CL1) in this brain region [72]. These neuroinflammatory events were associated with an increase in anxiogenic-related behavioral responses [72–74]. Interestingly, mice lacking TLR4 or TLR2 receptors protect against ethanol-induced cytokine and chemokine release and associated behavioral effects during alcohol abstinence [72].

Most chemokines assayed in our study, particularly MCP-1/CCL2, MIP-1α/CCL3 and MIP-1β/CCL4, appear to have essential implications in either triggering or modulating mood-related disorders, as demonstrated for functional chemokines such as, MCP-1/CCL2, MIP-1α/CCL3, and IP-10/CXCL10 [75–77]. These chemokines have been implicated in the modifications of neurotransmission and alterations in cognitive function [75–77], while CXCL8 and CXCL10 were found associated with alterations in neuroendocrine regulation and hypothalamic–pituitary–adrenal axis function [21, 33, 65]. The increased chemokine levels shown herein may reflect the activation of the inflammatory response system in women exhibiting severe anxiety and depression during pregnancy [13, 15, 21].

It is well known that Th1/Th2 and Th17/Treg immune balances are needed to maintain a successful pregnancy [12] and thereby of the conceptus [78]. It has been shown that pregnant patients with MDD display a preferential Th1-inflammatory response [59]. Recent studies from our group showed that serum levels of Th1, Th2 and Th17-related cytokines in pregnant subjects displaying affective disorders correlated with HARS and HDRS scores [15], and in a similar fashion shown herein, chemokines such as, MIP-1α/CCL3, MIP-1β/CCL4, MCP-1/CCL2, TARC/CCL17, Eotaxin/CCL11, and IL-8/CXCL8-related chemokines showed positive correlations with either HARS or HDRS scores in our pregnant population exhibiting ANX and/or ANX + DEP, respectively. These data posit that chemotactic cytokine appear to impinge at different levels on both neurons and glia cells [24], which ultimately leads to the deregulation of distinct neurotransmission systems shown to be implicated in mood-related disorders) [51, 79], in cognitive functions [21, 22] and in the functional activity of the HPA axis [76, 77].

Moreover, most serum chemokines assayed in the present study were conspicuously increased in women exhibiting mixed anxiety and depression compared with pregnant women only displaying anxiety. These observations support that these disorders during pregnancy represent a highly complex pathological condition associated with several biological responses, such as the increase in cortisol levels [14], glucocorticoid resistance, and inflammatory response [51].

Previous studies showed that BMI significantly correlated with pro-inflammatory cytokines in depressed patients with obesity [52] and pregnant women exhibiting severe anxiety and comorbid depression [15]. However, our results showed that only MCP-1/CCL2 and RANTES/CCL5 were found associated with BMI in pregnant women with ANX, but not in women in women exhibiting ANX + DEP (Table 4). This suggests that such chemokines should be related with inflammatory conditions in patients exhibiting high stress and anxiety symptomology, as previously reported for the increased serum concentrations of Th1/Th17- related cytokines in women with affective disorders [15].

Our results support other clinical studies, that showed that maternal IL-6 and IL-8 correlated with BMI and maternal adiposity in pregnant non-diabetic women during the third trimester of pregnancy [53]. A similar correlation was also observed with high levels of serum and follicular fluid MCP-1 concentrations in obese women, when compared to either overweight and normal-weight women [58], or with MCP-1, and high-molecular-weight (HMW) adiponectin and FFA (free fatty acid) /albumin in women displaying severe pregnancy-induced hypertension [80].

Thus, it appears that chemokines might be linked to women with pathological conditions, in the pregestational period throughout pregnancy, or in the postpartum. For instance, recent studies showed that the tumor necrosis factor ligand superfamily member (TRANCE), the hepatocyte growth factor, IL-18, the Fibroblast Growth Factor (FGF)-23, and CXCL1 were found significantly elevated in women with postpartum depressive symptoms; suggesting that these women show a compromised adaptability of the immune system [81].

Our data suggest that body mass measures (weight < 70 kg, BMI < 30 kg/m^2^) represent important variables to consider when evaluating depressive symptoms in women from mid to late pregnancy [82], particularly in women exhibiting increased stress responsiveness with high levels of anxiety [62].

Interestingly, different studies revealed that a decrease in chemokine levels (MCP-1, MIP-1α) during the first half of pregnancy is related to the physiological reduction in immune activity and leukocyte migration [83]. Increased levels of serum MIP-1α and decreased levels of MCP-1 in the first trimester were found associated with the development of preeclampsia, premature labor, and neonate-low birth weight [83].

Moreover, our results showed that serum levels of chemokines such as IL-8/CXCL8, MIP-1β/CCL4, MCP-1/CCL2, and Eotaxin/CCL11, remained elevated in both symptomatic groups, after controlling clinical measures by specific confounders; suggesting that such functional chemokines should enhance or interact with several other immune (i.e., Th1-related cytokines) and/or non-immune mediators (i.e., monoamines, prostaglandins, neurosteroids) in limbic structures [5, 12], regulating the intensity of anxiety and depressive symptoms, as demonstrated for Th1/Th17 cytokines in pregnant women with high levels of anxiety and severe depression [15].

Thus, the role of chemokines and their cognate receptors need to be explored deeply in psychiatric disorders in both pregnant and non-pregnant subjects, encompassing their signaling systems on the activated immune (NK, MΦ) cells.

## Conclusions and perspectives

Our data show that serum levels of most chemokines are increased in women exhibiting intense affective symptoms during late pregnancy. Our results posit that ANX, affective symptoms, and mood-related disorders may promote changes in immune mediators and their receptors leading to chronic inflammatory processes over time. If not controlled, it may enhance adverse obstetric and negative neonate outcomes, changes in child development and behaviors, and neuropsychiatric disorders.

### Limitations

Several limitations found in the present study included the cross-sectional design of the project and not a longitudinal one from early pregnancy to postpartum to detect changes in the chemokine profile at each trimester and in the early postpartum in our recruited population.

Data were collected in women displaying moderate to severe affective symptoms, and the study excluded participants with a history of smoking and alcohol consumption. This group could have shed important information and data in the present study.

Also, the study required the measurements of chemokine concentrations in pregnant women exhibiting moderate to severe depression without anxiety. Moreover, pregestational women displaying affective symptoms could extend the knowledge about the relevance of these disorders before pregnancy and their relation with the serum levels of chemokines, as depicted herein.

## Data Availability

All datasets used or analyzed during the current study are available from the corresponding author on reasonable request.

## Abbreviations

gwk: Gestation weeks
HARS: Hamilton Anxiety Rating Scale
HDRS: Hamilton Depression Rating Scale
DSM-5: Diagnostic and Statistical Manual of Mental Disorders
CTRL: Control
